# Immunotherapy and IVF Outcomes in Unexplained Recurrent Pregnancy Loss: A Systematic Review with Implications for Personalized Reproductive Medicine

**DOI:** 10.3390/jpm15120606

**Published:** 2025-12-06

**Authors:** Giosuè Giordano Incognito, Carla Ettore, Marco D’Asta, Ferdinando Antonio Gulino, Roberta Foti, Roberto Tozzi, Orazio De Tommasi, Pierluigi Chieppa, Stefano Di Michele, Giuseppe Ettore

**Affiliations:** 1 Obstetrics and Gynecology Unit, Maternal Child Department, ARNAS Garibaldi Nesima, 95122 Catania, Italy; cettore@arnasgaribaldi.it (C.E.); mdasta@arnasgaribaldi.it (M.D.); gettore@arnasgaribaldi.it (G.E.); 2 Unit of Gynecology and Obstetrics, Department of Human Pathology of Adults and Developmental Age, “G. Martino” University Hospital, 98125 Messina, Italy; ferdinandoantonio.gulino@unime.it; 3 Division of Rheumatology, AOU Policlinico “G. Rodolico”-San Marco, 95123 Catania, Italy; robertafoti@hotmail.it; 4 Unit of Gynecology and Obstetrics, Department of Women and Children’s Health, University of Padua, 35128 Padua, Italy; roberto.tozzi@unipd.it (R.T.); orazio.detommasi@studenti.unipd.it (O.D.T.); 5 Gynecology and Obstetrics 1, Department of Surgical Sciences, A.O.U. City of Health and Science of Turin, S. Anna Hospital, 10126 Turin, Italy; pierluigi.chieppa@unito.it; 6 Division of Obstetrics and Gynecology, Department of Surgical Sciences, University of Cagliari, 09124 Cagliari, Italy; dr.dimichelestefano@gmail.com

**Keywords:** immunology, embryo implantation, assisted reproductive technique, in vitro fertilization, recurrent pregnancy loss

## Abstract

**Background/Objectives:** Recurrent pregnancy loss (RPL) is one of the most challenging conditions in reproductive medicine, particularly when no identifiable cause can be determined after diagnostic evaluation. Although the role of immunological dysregulation has been hypothesized, the implementation of immunotherapies in clinical practice is controversial due to inconsistent findings and methodological heterogeneity across studies. This systematic review aims to provide an overview of the main characteristics of existing research on the role of immunological interventions in relation to In Vitro Fertilization (IVF) outcomes in women with RPL. Given the marked inter-individual variability in immunological mechanisms among affected women, evaluating these treatments may help identify future directions for personalized reproductive medicine. **Methods:** A comprehensive bibliographic search was systematically conducted from inception to October 2025 across databases, including Medline, Embase, Scopus, the Cochrane Database of Systematic Reviews, and ClinicalTrials.gov. Studies were included if they evaluated the efficacy of immunological treatments in women with unexplained RPL, comparing IVF outcomes between case and control groups. **Results:** Six cohort studies were included, four retrospective and two prospective. The immunological treatments investigated were granulocyte colony-stimulating factor (G-CSF), intravenous intralipid (with or without prednisolone), and lymphocyte immunization therapy (LIT). Despite some promising results, particularly for G-CSF and LIT, the studies were limited by small sample sizes, heterogeneous diagnostic criteria for RPL, and inconsistent treatment protocols. Furthermore, not all IVF outcomes, such as implantation and biochemical pregnancy rates, were reported. **Conclusions:** Current evidence is insufficient to support the use of immunotherapy in clinical practice for improving IVF outcomes in women with unexplained RPL. The variability in study design, patient selection, and immunotherapy regimens hinders the ability to draw firm conclusions. Well-designed randomized controlled trials with standardized definitions and outcome measures are needed to determine whether and for whom immunological treatments may offer clinical benefit.

## 1. Introduction

Pregnancy loss (PL) is defined as the spontaneous demise of a pregnancy before fetal viability, generally considered to occur before 24 weeks of gestation. However, this threshold may vary depending on national and institutional guidelines. Recurrent pregnancy loss (RPL) is typically defined as the occurrence of two or more PL. It is further classified as primary RPL in women with no previous viable pregnancy beyond 24 weeks, and secondary RPL when the losses follow one or more prior pregnancies that reached viability [1]. The optimal definition remains debated, with some guidelines adopting a stricter threshold of three or more consecutive losses [2]. Estimating the true prevalence of RPL is challenging, though it is believed to affect approximately 1–5% of couples of reproductive age attempting conception [3]. This variability is partially explained by the lack of uniform diagnostic criteria and the complexity of contributing factors. Indeed, reproductive outcomes after In Vitro Fertilization (IVF) are influenced by a multitude of elements, including maternal age, endocrine and uterine status, embryo quality, male factor infertility, and even clinic protocols, laboratory expertise, and regional regulatory frameworks. Currently, the diagnostic work-up recommended by evidence-based management guidelines identifies an underlying cause in approximately 50% of RPL cases [1]. The most frequently identified causes include embryonic chromosomal abnormalities, either de novo or linked to parental karyotypic rearrangements such as reciprocal or Robertsonian translocations [4]; infectious etiologies [5]; endocrine disorders such as thyroid dysfunction, diabetes, or polycystic ovary syndrome [6]; uterine anomalies [7]; antiphospholipid syndrome and other autoimmune conditions [8]; thrombophilia [9]; and sickle cell disease [10].

Nevertheless, in nearly half of all cases, no cause is identified, leading to the diagnosis of unexplained RPL [1]. This diagnostic uncertainty, combined with heterogeneity in terminology and clinical presentation, makes RPL a significant challenge in assisted reproductive technologies (ART). As a result, many diagnostic and therapeutic interventions are applied in practice, often empirically and without robust evidence. Among the conditions universally agreed upon as requiring investigation in cases of RPL are uterine malformations, antiphospholipid syndrome, and thyroid dysfunction [11]. However, some authors advocate for broader work-ups, including the evaluation of hereditary thrombophilia, antinuclear antibodies (ANA), and sperm quality, though these remain controversial and are not uniformly endorsed [12].

In particular, immunological factors have been postulated as key contributors to RPL, especially when conventional etiologies are excluded [13]. Women with RPL have shown specific immune alterations, including increased uterine NK cell activity and unfavorable KIR–HLA-C combinations in the innate immune system, as well as reduced regulatory T-cell activity and a Th1-skewed cytokine profile in the adaptive immune system. Despite growing interest, routine immunological screening is not recommended, as no biomarker, including NK activity, cytokine ratios, or T-cell subsets, has shown sufficient predictive accuracy for clinical use [14]. Despite these findings, the absence of standardized immunological diagnostic pathways complicates the clinical translation of such evidence. In this context, immunotherapies have emerged as a proposed treatment option for improving pregnancy outcomes in women with unexplained RPL. Given the heterogeneity of immunological profiles in unexplained RPL, evaluating targeted immunotherapies may contribute to the development of personalized reproductive medicine approaches tailored to specific patient immune characteristics.

Therefore, this systematic review aims to provide a comprehensive overview of the available evidence on immunotherapeutic interventions and their potential to improve IVF outcomes in patients with unexplained RPL.

## 2. Materials and Methods

This systematic review was conducted in accordance with the Preferred Reporting Items for Systematic Reviews and Meta-Analyses (PRISMA) guidelines [15]. The protocol was not registered in any registry. The PRISMA checklist used for reporting this review is available as Supplementary Material (Supplementary Table S1). Since only published, de-identified data were included, the study was exempt from institutional review board approval.

The review was restricted to published original research articles evaluating the effect of various immunotherapies in women with unexplained RPL undergoing IVF. Studies were eligible if they reported and compared IVF outcomes, specifically implantation rate (IR), biochemical pregnancy rate (BPR), clinical pregnancy rate (CPR), ongoing pregnancy rate (OPR), miscarriage rate (MR), and live birth rate (LBR), between cases and controls.

A comprehensive and systematic bibliographic search spanning from inception to October 2025 was performed across databases, including Medline, Embase, Scopus, the Cochrane Database of Systematic Reviews, and ClinicalTrials.gov. The first search strategy included the following combination of terms: (“immunotherapy” OR “immunological therapy” OR “immunological treatment” OR “immunological intervention” OR “immunologic therapy” OR “immunologic treatment” OR “immunologic intervention”) AND (“recurrent pregnancy loss” OR “RPL” OR “recurrent spontaneous abortion” OR “RSA” OR “recurrent miscarriage”) AND (“assisted reproductive technology” OR “ART” OR “in vitro fertilization” OR “IVF” OR “intracytoplasmic sperm injection” OR “ICSI”). A second search was carried out by combining each immunotherapy identified in the initial search AND (“recurrent pregnancy loss” OR “RPL” OR “recurrent spontaneous abortion” OR “RSA” OR “recurrent miscarriage”) AND (“assisted reproductive technology” OR “ART” OR “in vitro fertilization” OR “IVF” OR “intracytoplasmic sperm injection” OR “ICSI”).

**Figure 1:**
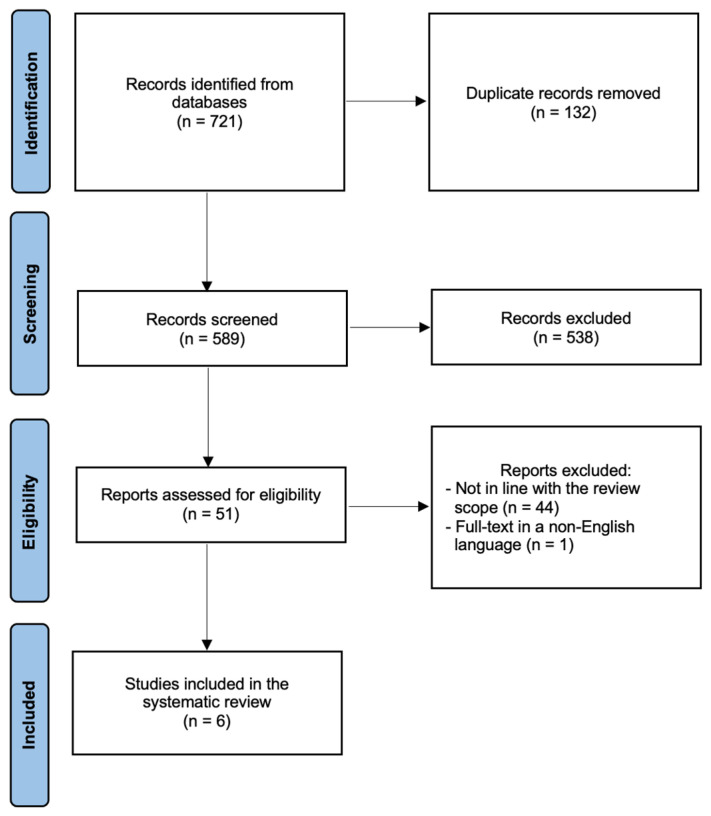
PRISMA flow diagram.

Only studies involving human participants and published in English were considered. Case reports, case series, commentaries, letters to editors, editorials, reviews, and conference abstracts were excluded. The PRISMA flow diagram (Figure 1) summarizes the search strategy.

All retrieved records were uploaded into the Rayyan platform (Qatar Computing Research Institute, rayyan.qcri.org, accessed on 31 October 2025) [16]. Two authors (G.G.I. and C.E.) independently screened titles and abstracts to identify studies for full-text review. Consensus on the relevance was reached by mutual agreement. In case of opinion discrepancy, studies were discussed with a third investigator (G.E.). Full-text articles were independently assessed by two authors (G.G.I. and C.E.), who extracted the relevant data using standardized data extraction forms. Any disagreements were resolved through discussion with the remaining authors, and a final abstraction form was compiled accordingly. Additionally, the reference lists of all known primary articles and relevant reviews were manually retrieved to identify any additional eligible studies not captured by the electronic searches. Corresponding authors were contacted for clarification in cases of unclear or missing information.

For each included study, the following data were recorded: type of immunotherapy, authors and year of publication, country, study design, study period, inclusion criteria, sample size (case and control groups), details of the intervention and control protocols, any co-administered medications, and IVF outcomes.

## 3. Results

Figure 1 summarizes the process of literature identification and selection of the studies. The systematic bibliographic research strategy identified a total of 721 studies, of which 132 duplicates were removed. After screening titles and abstracts, 51 full-text articles were assessed for eligibility. Ultimately, 6 studies met the inclusion criteria and were included in the systematic review [17,18,19,20,21,22]. The main characteristics of these studies are summarized in Table 1.

The immunotherapies investigated included granulocyte colony-stimulating factor (G-CSF) [17], intravenous intralipid, administered either as a standalone therapy [18,19] or in combination with prednisolone [20], and lymphocyte immunization therapy (LIT) [21,22]. The selected studies were published between 2013 and 2021. Three were conducted in Europe, respectively, in Germany [17], France [19], and Ireland [20], one in the United States [18], one in China [21], and one in Argentina [22]. All were cohort studies; four were retrospective [17,18,19,22] and two were prospective [20,21]. Concerning the definition of RPL, three studies (50%) defined RPL as two or more PL [17,20,22], while one adopted a broader definition of RPL as one or more PL [18], and two required three or more PL [19,21]. Notably, two studies included maternal age as a selection criterion, specifically women aged 40–42 years [18] or younger than 42 years [20]. In total, the six studies included 663 participants: 347 received immunotherapy and 362 served as controls. Forty-six women were included in both groups at different points in time under differing protocols. In all studies, the control group consisted of patients who did not receive any non-standard therapies. In one study [17], an additional control group comprised women receiving non-immunological interventions. Additional medications such as folic acid, progesterone, low-dose aspirin, low-molecular-weight heparin (LMWH), prednisolone, vitamin D, omega-3 supplements, and B complex vitamins were administered in three studies [17,19,20], whereas in the remaining three [18,21,22], patients did not receive any adjunctive therapies beyond standard IVF protocols. The only IVF outcomes evaluated across the studies were CPR [17,18,21], MR [20,21], and LBR [17,18,19,21,22]. None of the included studies reported IR, BPR, or OPR data. Among the interventions, G-CSF administered from the day of embryo transfer up to 12 weeks of gestation was associated with favorable outcomes in terms of both CPR and LBR in the treated group compared with controls [17]. Findings regarding intravenous intralipid therapy were heterogeneous. Intralipid was administered during the mid-follicular phase [18], on day 8 of the embryo transfer cycle with subsequent infusions at 3, 5, and 9 weeks of gestation [19], or one week before and after embryo transfer with a third dose after pregnancy confirmation [20]. About IVF outcomes, two studies [19,20] demonstrated positive effects of intralipid administration, of which one [20] was in the context of a combined immunomodulatory protocol including prednisolone initiated at the start of controlled ovarian stimulation or endometrial preparation and continued until 12 weeks of gestation. In contrast, another paper [18] did not report any benefit in terms of LBR following intralipid therapy. Similarly, the administration protocols for LIT differed among the included studies. In one study, LIT was administered every two weeks for four sessions, followed by monthly administration for an additional four sessions [21]. In the other paper, it was administered every two weeks [22]. Both studies reported improved IVF outcomes.

## 4. Discussion

The limited available literature is marked by significant methodological heterogeneity and yields conflicting results concerning the efficacy of immunotherapies in improving IVF outcomes among women with unexplained RPL. Several factors contribute to the difficulty in interpreting the data. One of the most significant is the lack of uniformity in the definition of RPL across studies, which leads to heterogeneous study populations. Moreover, the studies included a limited number of participants, reducing statistical power, and often applied immunological treatments without basing them on preliminary diagnostic assessments or immune profiling. The inconsistency in pre-ART fertility evaluations across studies further exacerbates this variability, complicating comparisons of outcomes. Another major limitation is the absence of standardized protocols for the immunotherapies assessed in more studies. Variations in dosage, timing, and duration of administration were frequent and rarely justified based on individual patient characteristics or immunological findings. Consequently, although some findings appear promising, none of the interventions evaluated can currently be recommended for routine clinical use without further, more rigorous research. These limitations underscore the need to adopt an evidence-based approach to managing RPL, a condition that is not only complex and multifactorial but also highly individualized. RPL should not be considered a single pathological entity with rigid diagnostic criteria, but rather a clinical manifestation that may differ significantly across patients and clinical contexts, especially in the ART setting. This perspective highlights the importance of tailoring therapeutic strategies to individual patients. Further research is needed to identify specific immunological dysfunctions associated with RPL and evaluate the effectiveness of targeted therapies. Establishing clear diagnostic criteria and standardized treatment protocols would improve study comparability and allow for more reliable meta-analyses. Prioritizing investigations into the immunological underpinnings of RPL may enable more precise, individualized interventions. In the interim, clinicians must carefully balance the need for evidence-based practice with the need for personalized patient care. Until more definitive evidence becomes available, managing unexplained RPL should remain cautious, avoiding the routine use of immunotherapies outside of research settings. The most recent ESHRE guideline on RPL emphasizes the importance of a pragmatic, empathetic approach to managing this condition, tailored to each patient’s unique clinical context [1].

The immunotherapies identified in the present systematic review are discussed below.

### 4.1. Granulocyte Colony-Stimulating Factor

#### 4.1.1. Mechanism of Action

G-CSF is a glycoprotein cytokine that plays a central role in hematopoiesis by stimulating the proliferation, differentiation, and activation of neutrophilic granulocyte precursors within the bone marrow [23]. It is produced by various cell types, including stromal cells, fibroblasts, endothelial cells, monocytes, macrophages, and bone marrow-derived cells. Its biological activity is mediated through binding to a specific receptor, expressed not only on hematopoietic lineage cells, such as myeloid progenitors, mature neutrophils, monocytes, some lymphocyte subtypes, and platelets, but also on non-hematopoietic cells, including vascular endothelial cells, placental cells, trophoblasts, and luteinized granulosa cells [24]. These pleiotropic receptor distributions support the hypothesis that G-CSF may exert immunomodulatory, angiogenic, and tissue-supportive effects at the maternal-fetal interface. In experimental models, G-CSF has been shown to enhance trophoblast growth and support placental metabolic activity, suggesting a potential role in early implantation and maintenance of pregnancy [25].

#### 4.1.2. Efficacy

A retrospective cohort study conducted by Santjohanser et al. [17] investigated the effects of G-CSF in women with a history of at least two early PL undergoing IVF. A total of 199 IVF cycles in 127 women were analyzed and divided into three groups: one receiving G-CSF (49 patients), one without any adjunctive treatment (33 patients), and one receiving alternative non-immunological medications (45 patients). In the G-CSF group, 11 patients received 34 × 10^6^ IU once weekly and 38 patients received 13 × 10^6^ IU twice weekly, starting from the day of embryo transfer and continuing through the 12th gestational week. The “other medications” group was treated with various agents, including enoxaparin (40 mg/day), acetylsalicylic acid (100 mg/day), folic acid (5 mg/day), corticosteroids (prednisone 2.5–5.0 mg/day or dexamethasone 0.5 mg/day), and doxycycline (100 mg/day for five days, starting at embryo transfer). All participants received folic acid (0.5 mg/day) and luteal phase support with vaginal progesterone (200 mg three times daily until week 12 of gestation). The group treated with G-CSF showed improved reproductive outcomes, with a CPR of 47% and an LBR of 32%. In comparison, the CPR and LBR were 27% and 14%, respectively, in the group treated with other medications (*p* = 0.016 and *p* = 0.006), and 24% and 13% in the untreated group (*p* = 0.016 for both comparisons). Despite these encouraging results, several methodological limitations must be acknowledged. The retrospective design, the inclusion of multiple IVF cycles per patient, and the lack of clarity regarding whether CPR and LBR were calculated per cycle or per patient limit the generalizability of the findings. Additionally, key prognostic variables were not equally distributed across the groups, potentially introducing selection bias.

#### 4.1.3. Safety

Data on the safety of G-CSF use during pregnancy are reassuring. Among 35 women treated with G-CSF during the first trimester, one developed a transient skin rash, and two experienced leukocytosis (>25,000/mm^3^). No congenital anomalies or developmental abnormalities were observed in the newborns, all of whom exhibited expected perinatal outcomes [26]. A larger safety analysis comparing 100 pregnancies exposed to G-CSF with 124 unexposed controls found no significant differences in obstetric or neonatal complications [27]. These findings suggest that G-CSF may be a safe adjunct in early pregnancy; however, further studies with larger populations and longer follow-ups are needed to confirm its safety profile conclusively.

### 4.2. Intravenous Intralipid

#### 4.2.1. Mechanism of Action

Intralipids are a fat emulsion composed of soy oil, glycerine, and egg phospholipids, initially developed for parenteral nutrition in patients unable to maintain adequate oral intake. The rationale for their use in reproductive medicine emerged from experimental animal models. In a mouse model of partner-specific recurrent abortion, intraperitoneal administration of 20% intralipid during early pregnancy reduced the MR from 38% (43/112) in untreated mice to 11% (9/83, 95% CI = 4.3–17.7%). The initial proposal to use lipid emulsions in the treatment of RPL was based on animal data showing reduced fetal resorption rates in specific mouse mating models [28]. In human studies, intravenous intralipid has demonstrated immunomodulatory potential through several mechanisms: inhibition of natural killer (NK) cell cytotoxicity [19], impairment of macrophage antigen presentation function [29], and reduction in NK and lymphokine-activated killer (LAK) cell activity [30]. Additionally, it may suppress the secretion of pro-inflammatory cytokines produced by Th1 cells [31].

#### 4.2.2. Efficacy

Three studies included in this review investigated the efficacy of intravenous intralipid in improving IVF outcomes in patients with unexplained RPL. A retrospective cohort study by Check et al. [18] evaluated women aged 40–42 years with a history of miscarriage or previous IVF failure. Ten patients received intravenous intralipid (4 mL of 20% solution diluted in 100 mL saline over one hour) during the mid-follicular phase, and the results were evaluated after ten matched cycles. No clinical pregnancies occurred in this group, whereas the control group had a 40% CPR and a 30% LBR (*p* = 0.087). Due to these preliminary findings, the study was terminated early. Opposite conclusions were reported in a retrospective cohort study by Plaçais et al. [19]. Women with at least three first-trimester PL and no identifiable cause, such as uterine anomalies, genetic, infectious, hormonal, thrombophilic, or autoimmune conditions, were included. Ten women received intralipid infusions on day 8 of the embryo transfer cycle and again at 3, 5, and 9 weeks of amenorrhea in the case of ongoing pregnancy. The therapy was selected in cases of suspected endometrial immune overactivation, failed steroid therapy, or overweight patients who could not receive steroids. Each patient was matched with two controls. Concomitant medications included low-dose aspirin, LMWH, prednisone (10 mg/day), progesterone, and vitamin D. A LBR of 70% was reported in the intralipid group, significantly higher than the 15% observed in controls (*p* = 0.02). A longitudinal prospective cohort study by Harrity et al. [20] included 46 women under 42 years old with two or more previous miscarriages before 22 weeks following the transfer of good-quality embryos. All participants had normal endometrial cavity anatomy and endocrine profile (thyroid and prolactin levels within the normal range). CD4+ intracellular cytokine ratios (CKR) were assessed, and those with elevated ratios were offered additional immunotherapy before their next ART cycle. Stimulated intracellular cytokine expression in peripheral CD4+ T cells has been linked to increased incidence of RPL [32], likely due to activation of cytotoxic NK cells via KIR receptors [33]. The immunotherapy protocol included intravenous intralipid infusions (20%) administered one week before and one week after embryo transfer, with a third dose after pregnancy confirmation, along with prednisolone (15–25 mg/day) starting from ovarian stimulation or endometrial preparation until 12 gestational weeks or pregnancy failure. Some patients also received a nutritional support regimen consisting of omega-3 (3 g/day), B-complex, and vitamin D3 (25 μg/1000 IU daily). A significant reduction in MR was observed in post-treatment ART cycles (39.4% vs. 92.6%, *p* < 0.0001).

#### 4.2.3. Safety

No serious adverse effects have been reported following the use of low-dose intralipid in women with RPL. However, adverse events have been documented with higher doses of intravenous lipid emulsions, including acute kidney injury, cardiac arrest, acute lung injury, venous thromboembolism, fat embolism, fat overload syndrome, pancreatitis, allergic reactions, and increased susceptibility to infections [34].

### 4.3. Prednisolone

#### 4.3.1. Mechanism of Action

Prednisolone is a synthetic glucocorticoid with anti-inflammatory and immunosuppressive properties. It is widely used in clinical practice to treat inflammatory and autoimmune diseases [35]. Its potential application in women with RPL of suspected immune origin is based on its ability to promote maternal-fetal immune tolerance through various mechanisms. Elevated levels of uterine NK cells have been associated with recurrent miscarriage. In women with preconceptional uterine NK cells above 5%, prednisolone administration significantly reduced their levels from 14% to 9% (*p* = 0.0004) [36]. Increased vascular maturation in the endometrium has also been observed in women with RPL, and prednisolone was shown to decrease the proportion of mature endometrial blood vessels, suggesting an additional role in modulating endometrial angiogenesis [37]. Other proposed mechanisms include increased regulatory T cells, suppression of Th1 cytokine production by T cells, and inhibition of NK cell cytotoxicity [38]. However, these effects require further confirmation in the specific context of RPL. Additional support for corticosteroid use in reproductive immunology comes from in vitro studies demonstrating that glucocorticoids can up-regulate HLA-G expression in first-trimester trophoblast cells, a molecule crucial for maternal immune tolerance and often dysregulated in women with recurrent miscarriage [39].

#### 4.3.2. Efficacy

The only study included in this review that assessed the impact of prednisolone on IVF outcomes in women with unexplained RPL was conducted by Harrity et al. [20], as previously detailed in the section on intravenous intralipid, with which prednisolone was administered as part of a combined immunomodulatory protocol.

#### 4.3.3. Safety

Potential adverse effects of corticosteroids in pregnancy include gestational hypertension, diabetes, preterm birth, and low birth weight when used at high doses. However, such complications are uncommon with low-dose prednisone (<10 mg/day) [40], as approximately 90% of the drug is metabolized by the placenta and only a small amount reaches the fetus in active form [41].

Several studies have evaluated the risk of congenital malformations associated with corticosteroid use in the first trimester and generally report no increased risk. The only debated concern has been a possible association with oral clefts, though most retrospective and prospective case–control studies have not confirmed this link [40,42].

### 4.4. Lymphocyte Immunization Therapy

#### 4.4.1. Mechanism of Action

LIT was the first immunological treatment proposed for couples with RPL. In 1981, Taylor and Faulk [43] described successful pregnancies in three women with a history of RPL who were treated with leukocyte-rich plasma from unrelated donors based on the immunosuppressive theory of fetal tolerance. The use of allogeneic LIT gained broader attention following a randomized controlled trial published in 1985 [44], which reported improved LBR in women with RPL who received immunization with paternal lymphocytes. However, a major turning point occurred with the publication of the REMIS study [45], a double-blind, multicenter, randomized trial that found poorer outcomes in women treated with LIT compared with controls. Based on these findings, the U.S. Food and Drug Administration (FDA) ruled in 2002 that immunization with partner lymphocytes could only be performed in clinical trials. Nevertheless, many subsequent studies criticized the REMIS trial for methodological limitations. The treatment involved a single administration of mononuclear cells, mostly intravenously, a route considered low in immunogenicity and limited to intradermal injections. In contrast, protocols associated with better outcomes typically used multiple intradermal doses before conception, often in combination with pregnancy immunotherapy [46]. Indeed, a meta-analysis by Liu et al. [47] demonstrated higher success rates when LIT was administered both before and during pregnancy. Additional concerns were raised over the REMIS protocol itself. The mononuclear cells were stored overnight at 4 °C before administration, a practice shown to reduce CD200 expression on antigen-presenting cells, potentially impairing immunological efficacy [48]. Moreover, patients with anti-nuclear antibodies, who are known to have a higher risk of miscarriage [49], were not excluded. The theoretical rationale for LIT lies in the hypothesis that women with RPL may lack protective anti-paternal antibodies or “blocking antibodies” that shield the fetus from maternal immune rejection. LIT is intended to induce these antibodies [50]. However, while many trials selected patients based on the absence of these antibodies, the clinical relevance of anti-paternal antibodies remains uncertain [51]. Several variables may influence LIT efficacy, including cell preparation, injection route, administration frequency, and patient immunologic profile (e.g., autoimmune disorders), underscoring the importance of careful patient selection [52].

#### 4.4.2. Efficacy

Two studies included in this review evaluated the efficacy of LIT in improving IVF outcomes in patients with unexplained RPL. Chen et al. [21] enrolled women with more than two early PL and excluded those with abnormal immune function, endocrine disorders, reproductive tract infections, abnormal karyotypes, or uterine anomalies. Among 288 patients, 134 received LIT. Twenty milliliters of peripheral blood were collected from the male partner, and 1 × 10^8^ mononuclear cells were isolated via Ficoll gradient, suspended in saline, and administered intradermally every two weeks for four doses. In cases of pregnancy, monthly maintenance doses were given for four additional sessions. The CPR was significantly higher in the treated group compared to controls (97.0% vs. 53.2%, *p* < 0.0001), with lower MR (8.5% vs. 18.3%, *p* < 0.05) and higher LBR (80.8% vs. 53.7%, *p* < 0.0001). Fainboim et al. [22] studied 152 women with more than three PL, normal karyotypes, and no autoimmune conditions. Ninety-eight received LIT. From the male partner, 120 mL of peripheral blood was collected; 1–2 × 10^8^ peripheral blood mononuclear cells (PBMCs) were obtained via Ficoll gradient, suspended in 1 mL of saline, and injected intradermally every two weeks. Treatment was continued until a mixed lymphocyte reaction blocking factor (MLR-Bf) above 50% was reached. MLR-Bfs are present in women who recently delivered a live infant and are considered biomarkers for pregnancy progression and effective immune tolerance [53].

#### 4.4.3. Safety

The use of allogeneic cells raises safety concerns. In transfusion medicine, lymphocytes are routinely depleted to minimize risks. Reported complications include neonatal alloimmune thrombocytopenia, red blood cell alloimmunization with potential for erythroblastosis fetalis, transmission of infectious agents such as hepatitis or HIV, and possible long-term increased risk of hematologic malignancies [54]. Nonetheless, when administered intradermally and before conception, LIT appears to be associated with a low risk of serious adverse events [55].

## 5. Conclusions

This systematic review highlights the scarcity and heterogeneity of available evidence on the use of immunotherapy in women with unexplained RPL undergoing IVF treatment. The small number of studies included, differences in study populations, variability in immunotherapy protocols, and limited sample sizes all contribute to the current lack of clarity in the medical literature. Based on the available data, immunotherapy should not be recommended in routine clinical practice to improve reproductive outcomes and should be restricted to research settings. Well-designed randomized controlled trials with appropriate patient selection are strongly needed to better define the potential role of immunomodulatory strategies in this complex clinical context. In this regard, identifying specific immunological phenotypes could be crucial to guide future personalized reproductive medicine approaches.

## Figures and Tables

**Table 1:** Characteristics of the studies included in the systematic review.

Immunotherapy	Author, Year	Country	Design	Period	Inclusion Criteria	Participants (Cases vs. Controls)	Intervention	Control	Additional Medications	CPR (*n*, %)	MR (*n*, %)	LBR (*n*, %)
G-CSF	Santjohanser et al., 2013 [17]	Germany	retrospective CS	2002–2010	≥2 PL	127 (49 vs. 33 vs. 45)	11 patients: G-CSF 34 × 10^6^ IU/week; 38 patients: G-CSF 2 × 13 × 10^6^ MU/week from ET until gestational 12 weeks	33 patients: no treatment; 45 patients: other medications, i.e., LMWH (40 mg/day), acetylsalicylic acid (100 mg/day), folic acid (5 mg/day) or prednisone/dexamethasone (2.5–5.0 mg/0.5 mg/day) from the middle of the previous cycle until the embryonic heartbeat, and doxycycline (100 mg/day for 5 days) from ET	folic acid (0.5 mg/day) and progesterone vaginally (3 × 200 mg/day in the luteal phase until 12 weeks)	34 (47%) vs 11 (24%) vs. 22 (27%) (↑)	NR	23 (32%) vs 6 (13%) vs. 11 (14%) (↑)
Intravenous intralipid	Check et al., 2016 [18]	USA	retrospective CS	NR	≥1 PL; age 40–42 years	20 (10 vs. 10)	intralipid (4 mL diluted at 20% in 100 mL saline) infusion over 1 h during the midfollicular phase	no treatment	none	0 (0%) vs. 4 (40%) (↓)	NR	0 (0%) vs. 3 (30%) (↓)
	Plaçais et al., 2020 [19]	France	retrospective CS	2015–2018	≥3 PL (<12 weeks)	30 (10 vs. 20)	intralipid infusion at day-8 of the cycle of ET, and then at 3, 5 and 9 gestational weeks	no treatment	low dose aspirin, LMWH, prednisolone (10 mg/day), progesterone, vitamin D	NR	NR	7 (70%) vs. 3 (15%) (↑)
Intravenous intralipid and Prednisolone	Harrity et al., 2018 [20]	Ireland	prospective CS	March 2013–March 2016	≥2 PL (<22 weeks); elevated CKR *, age < 42 years	46 (46 vs. 46 same group)	intralipid (20%) infusion 1 week pre- and 1 week post-ET and third dose after confirmation of pregnancy; prednisolone (15–25 mg) from COS or EP until 12 gestational weeks	no treatment	omega (3.3 g), B complex, vitamin D3 (25 μg/1000 iu) for ≈ 10 weeks, LMWH	NR	13 (39.4) vs. 137 (92.6%) (↓)	NR
LIT	Chen et al., 2020 [21]	China	prospective CS	January 2015–March 2019	≥3 PL	288 (134 vs. 154)	peripheral blood collected from the husband (20 mL), and 1 × 10^8^ monocytes obtained through a standard Ficoll gradient, suspended in saline solution, and injected intradermally every 2 weeks for 4 times and every month for 4 times	no treatment	none	130 (97.0%) vs. 82 (53.2%) (↑)	11 (8.5%) vs. 15 (18.3%) (↓)	105 (80.8%) vs. 44 (53.7%) (↑)
	Fainboim et al., 2021 [22]	Argentina	retrospective CS	January 2000–June 2018	≥2 PL	152 (98 vs. 54)	peripheral blood collected from the partner (120 mL), and 1–2 × 10^8^ PBMCs obtained through a standard Ficoll gradient, suspended in 1 mL of saline solution, and injected intradermally every 2 weeks until MLR-Bf > 50%	no treatment	none	NR	NR	60 (59%) vs. 20 (37%) (↑)

Abbreviations: CKR, CD4+ intracellular cytokine ratios; COS, controlled ovarian stimulation; CPR, clinical pregnancy rate; EP, endometrial preparation; ET, embryo transfer; G-CSF, granulocyte colony-stimulating factor; LBR, live birth rate; LIT, lymphocyte immunization therapy; LMWH, low molecular weight heparin; MLR-Bf, mixed lymphocyte reaction-blocking factor; MR, miscarriage rate; NR, not reported; PBMCs, peripheral blood mononuclear cells; PL, pregnancy loss. * defined as TNF-α/IL-10 > 30.6 and IFN-γ/IL-10 > 20.5. “↑” means the intervention is better than the control(s), “↓” means the intervention is less than the control(s).

## Data Availability

No new data were created or analyzed in this study.

## Supplementary Materials

The following supporting information can be downloaded at: https://www.mdpi.com/article/10.3390/jpm15120606/s1, Table S1: PRISMA 2020 checklist.
